# What Can Modeling Tell Us About Sustainable End Points for Neglected Tropical Diseases?

**DOI:** 10.1093/cid/ciab188

**Published:** 2021-06-14

**Authors:** Amanda Minter, Lorenzo Pellis, Graham F Medley, T Déirdre Hollingsworth

**Affiliations:** 1 Big Data Institute, Li Ka Shing Centre for Health Information and Discovery, University of Oxford, Oxford, United Kingdom; 2 Department of Mathematics, University of Manchester, Manchester, United Kingdom; 3 The Alan Turing Institute, London, United Kingdom; 4 Centre for Mathematical Modelling of Infectious Diseases, London School of Hygiene and Tropical Medicine, London, United Kingdom

**Keywords:** sustainable control, elimination, neglected tropical diseases

## Abstract

As programs move closer toward the World Health Organization (WHO) goals of reduction in morbidity, elimination as a public health problem or elimination of transmission, countries will be faced with planning the next stages of surveillance and control in low prevalence settings. Mathematical models of neglected tropical diseases (NTDs) will need to go beyond predicting the effect of different treatment programs on these goals and on to predicting whether the gains can be sustained. One of the most important challenges will be identifying the policy goal and the right constraints on interventions and surveillance over the long term, as a single policy option will not achieve all aims—for example, minimizing morbidity and minimizing costs cannot both be achieved. As NTDs move toward 2030 and beyond, more nuanced intervention choices will be informed by quantitative analyses which are adapted to national context.

Transmission dynamic modeling has been used in recent years to evaluate the effectiveness of ongoing and alternative treatment programs for reducing the global burden of the neglected tropical diseases (NTDs) [1, 2]. In late 2020, the World Health Organization (WHO) Roadmap [3] for NTDs was approved by the World Health Assembly and maps the way forward to 2030 with measurable targets for the reduction in morbidity and mortality of the NTDs. As programs move closer to these targets and toward ambitious more targets, the next phase in modeling NTDs will require predictions of indicators of resurgence, sustained transmission, and elimination alongside measures of cost, effort, and health outcomes.

The planning of longer term NTD programs will depend on the goal of elimination. This may be elimination of transmission, also referred to as interruption of transmission, which is achieved when there is zero incidence of the NTD in a defined geographic area [4]. Eradication is the permanent reduction to zero incidence. The feasibility of eradication for NTDs will be disease specific [5]. Similarly, WHO has defined disease specific goals, including, for several diseases defined as Elimination as a Public Health Problem (EPHP), meaning that the public health impact of these diseases has been reduced, even if transmission continues.

The feasibility of reaching and maintaining these goals will depend on the quality of the surveillance system in detecting new cases. For lymphatic filariasis, new cases have been found after an EPHP target threshold has been met [6], and there is evidence to suggest that a more intensive, and likely more costly surveillance threshold of <1% microfilaria (mf) prevalence, may not be enough to achieve elimination of transmission in some areas [7]. The 2030 target for schistosomiasis is EPHP [3], but modeling has shown that once morbidity or EPHP goals have been achieved, the goals may be maintained at lower efforts; however, there is also risk of resurgence in some settings where control efforts are reduced [8].

Most NTDs are characterized by very slow epidemic growth rates, but robust surveillance is required to ensure that cases are identified. To achieve greater certainty in prevalence or incidence, more effort, and hence more cost, is required. If such surveillance programs could identify increasing case numbers and subsequently aid in preventing resurgence, cases and costs would be averted in the future by triggering a public health response. However, for NTDs, this causes 2 challenges. First will there be the political will and budget available for responsive interventions, and can we calculate the cost of, for example, relaunching a locally targeted, effective mass drug administration in several years’ time? Second, as prevalence of NTDs becomes smaller, the cost of surveillance programs may increase to the extent where it may outweigh any cost of having sustained the intervention—for example, maintaining MDA versus surveillance to confirm that elimination has been achieved. The option then may not be an immediate goal elimination or eradication but a sustainable control system to maintain goals that have already been achieved. Such a system would have robust surveillance and response, due to the slow epidemic growth rates, that would be able to gradually achieve elimination and eradication but on a longer timescale and without the additional immediate investment to achieve elimination. The result would be a response that is manageable both in terms of cost and effort and more likely to be successful.

Transmission dynamic modeling can be used to evaluate the effectiveness of long-term surveillance programs in identifying likelihood or resurgence. The predicted performance of surveillance programs can be evaluated using model predictions while accounting diagnostic uncertainty, and these predictions can be combined with costs to develop models for sustainable control. In this article we discuss the steps toward developing such models and the challenges that accompany them (Figure 1).

**Figure 1.**
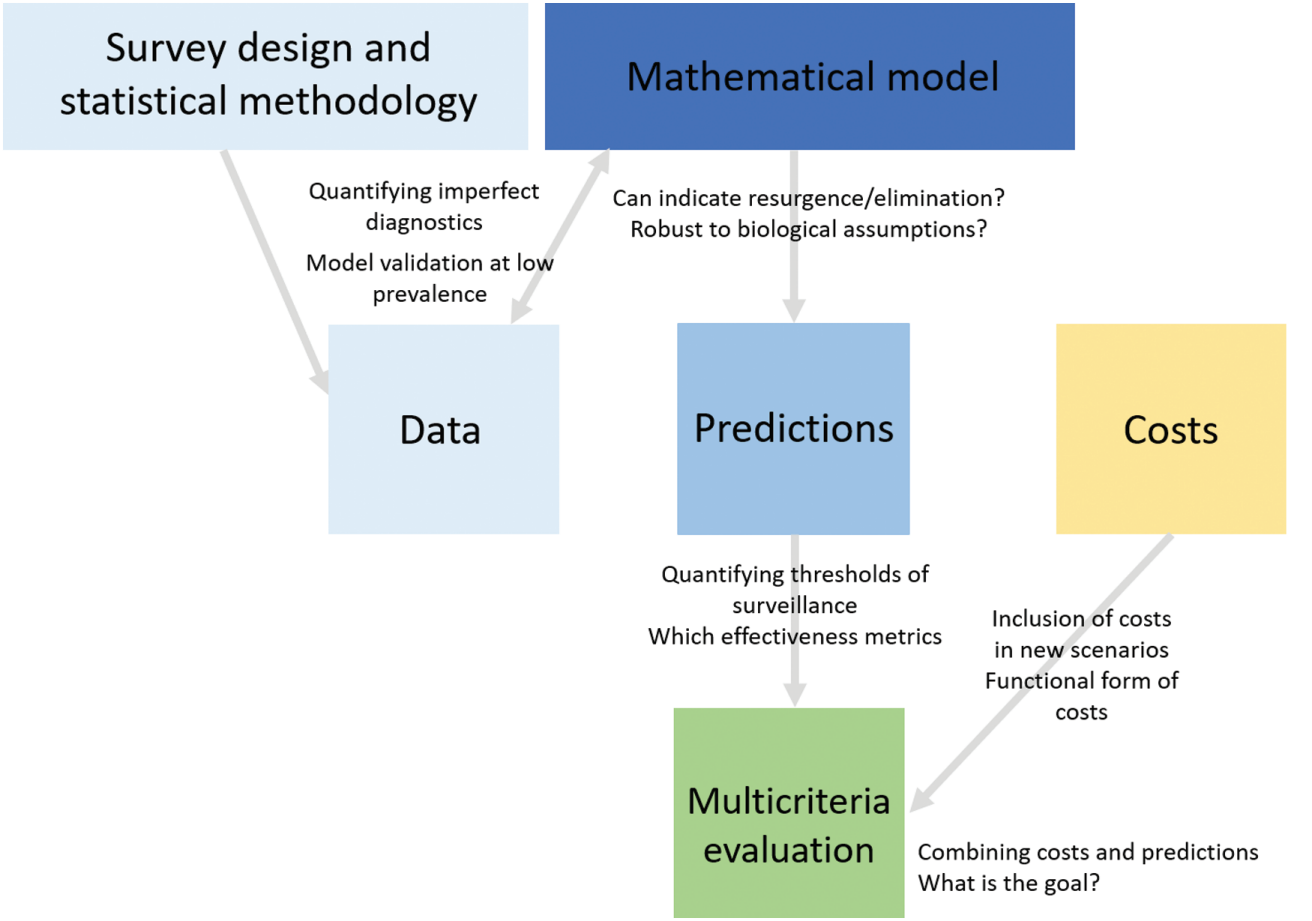
Conceptual framework describing the components of developing dynamic models and the respective challenges. The model is both informed by data and must predict data by accounting for diagnostic uncertainty. Model predictions and costs will inform the multicriteria evaluation of sustainable control programs.

## SURVEY DESIGN AND STATISTICAL METHODOLOGY

As programs move toward elimination, maximizing information given limited resources will aid in targeting treatment and identifying possible areas of resurgence. Who and when to sample in survey design should be informed by the duration of infectiousness and whether there are high-risk groups [9]. Research is needed to evaluate the effectiveness of potential alternative surveillance strategies. For example, research into understanding the capability of molecular xenomonitoring for detecting presence of lymphatic filariasis (LF) in human populations [10] and serology for visceral leishmaniasis (VL) in south Asia [11]. Additionally, exploring predictions at different geographic scales will aid in program planning; for example, one analysis suggests that the geographical scale of decision making affects the number of people that will require treatment for onchocerciasis [12].

Survey design must also be revaluated under the premise of limited resources, such as integrated surveillance [13], exploring survey designs, to optimize resource use in monitoring the drug efficacy for treating soil transmitted helminths and schistosomiasis [14] or utilising model-based geostatistics to optimise survey design and analysis [15]. Development of statistical tools will be complemented by mathematical models of underlying transmission dynamics.

## **CONNECTING MODELS TO DATA**: TRANSMISSION DYNAMICS AND IMPERFECT DATA

Mathematical models, once validated with data, can be used to predict infection dynamics after treatment programs have been stopped. These data, however, are a snapshot of underlying infection dynamics, arising from case detection of a subset of the population and often via imperfect diagnostic testing. Understanding the uncertainty arising from these observation processes will be critical to understanding whether elimination has truly occurred or whether treatment should be re-started. For example, recent model predictions of an increase in trachomatous inflammation–follicular (TF) prevalence after MDA has stopped could represent true resurgence or measurement error [16].

Imperfect diagnostic testing at low prevalence levels could also impact triggering of surveillance thresholds. A high number of false positives due to low specificity could result in a program being restarted incorrectly. Whereas a high number of false negatives due to low sensitivity could result in a program not being restarted, when in fact the program needs to be restarted. Models have been used to show that a decreased detection effort resulted in a decrease of observed VL incidence but an increase in the true incidence [17]. Mathematical models designed to connect predictions to data on the same scale and measure as the survey is designed to collect can be used to understand the identifiability of these outcomes and also to predict the impact on resurgence probability if the incorrect outcome is assumed to be true.

In addition, there is role for mathematical models in surveillance to assess the effectiveness of new diganostic tools or alternative control measures, such as vector control for LF [18]. Some diagnostic tests perform poorly in low transmission settings and so may not be suitable for in programs with a goal of elimination [19] whereas others may be useful for monitoring co-endemic areas [20]. In this collection, model-based assessments of the use of both microfilaremia and antigenemia data in predicting elimination of transmission of LF following MDA are presented. Models also have a role in evaluating the usefulness of new diagnostic tests in surveillance. For example, one analysis explores the relationship between occurrence of new VL cases and predicted biomarker prevalence, highlighting the potential role of population-based surveys for monitoring risk of VL resurgence [21].

For some NTDs, the underlying biological or epidemiological assumptions are not fully understood. The most informative age group for seromonitoring of onchocerciasis is influenced by the model assumptions of age-dependent exposure and the optimal threshold of seroprevalence for monitoring onchocerciasis transmission is influenced by model assumptions of density-dependent parasite establishment [22]. In addition, the model assumptions of the underlying biology and age-structured prevalence of soil-transmitted helminth affects the control effort required to reach the 2030 morbidity targets [23]. These model-based predictions were used to propose different program designs to account for such sources of uncertainty [23]. Understanding both structural uncertainty and observation uncertainty will be key to utilizing models to predict the dynamics of elimination.

## **CONNECTING MODELS TO PREDICTIONS**: **INDICATORS OF RESURGENCE AND EFFECTIVENESS METRICS**

Model predictions can be used to calculate effectiveness metrics to determine whether treatment can be reduced or stopped. Model-based thresholds of elimination can be calculated using either a deterministic model-based threshold, which if reached, elimination is certain (referred to as a critical threshold in [24]) or a stochastic model-based threshold. Stochastic model-based thresholds can be determined by calculating the certainty of elimination by using the proportion of eliminations detected by the threshold statistic that result in long-term eliminations [25]. Stochastic models have been used to determine model-based thresholds while accounting for diagnostic testing uncertainty [25].

Modeling has shown that resurgence can occur outside of the current recommended postintervention surveillance period for LF [26] and that higher coverage active screenings gives more certainty of elimination of transmission of gambiense human African trypanosomiasis in the future [27]. To achieve EPHP of *Schistosoma mansoni* and *Schistosoma haematobium*, community-wide treatment, instead of only treating school-aged children, is required in high transmission settings or in settings with low school enrollment [28]. In addition, the probability of sustaining the morbidity control goal of *S. mansoni* under different treatment strategies depends on transmission intensity and the level of adult burden [8].

Using prevalence alone is not always a sufficient measure of potential for onward transmission. Predicting the probability of elimination may be improved by using prevalence difference over multiple years instead of current prevalence only [26]. Although snapshots of TF prevalence do not directly relate to underlying transmissibility, TF prevalence over time can be used to detect hotspots of transmission that may benefit from increased treatment coverage [29].

It is important, however, to distinguish effectiveness metrics to minimize or maximize depending on whether we wish to monitor which subpopulations may contribute to onward transmission from the individuals with the greatest risk of morbidity. An appropriate metric to minimize onward transmission may be a particular age group: for example, the role of school-aged children in trachoma transmission [30] or individuals with high worm burden in schistosomiasis transmission [31]. However, these groups may not also have the highest risk of morbidity.

As many NTDs result in morbidity, disability-adjusted life years (DALYs) averted are commonly used as metrics of effectiveness [32]. However, DALYs may not always be an appropriate measure for all individuals. For example, DALYs of an average infection do not take into account the complicated burden of high intensity infections of schistosomiasis [31]. Turner et al [31] calculate 3 different effectiveness metrics that capture the effect of treatment for schistosomiasis on both parasite transmission (overall worm burden) and morbidity (prevalent infection case years averted and heavy case years averted). Different treatment programs cannot be simply planned according to simple effectiveness metrics of low, medium, or high prevalence; other disease- and setting-specific factors must be considered. Modelers will need to distinguish the metrics of transmission potential and the metrics of morbidity as we move towards models aimed at maintaining the gains.

## COMBINING COSTS AND PREDICTIONS FOR DECISION MAKING

As programs move toward low prevalence settings, countries will need to decide whether to continue to survey areas or stop surveillance. In these cases, they will also need to decide if they need to trigger the restart of treatment programs. Thresholds of active and passive surveillance after treatment has stopped will need to be defined. We can assume a passive surveillance threshold will be higher than any active surveillance threshold, but quantifying these thresholds will be challenging. Also, it is likely that in the timescale of NTD elimination, thresholds of detection may increase as diseases become rarer.

Given these surveillance thresholds, programs of active or passive surveillance will need to be costed. Unlike traditional cost-effective analysis, in models with sustainable control, costs will not remain constant over time. This can be achieved either through trying to reach final cases [32], restarting treatment programs, or implementation of new diagnostic tools [19]. Hence the total cost over a time horizon should be calculated [32], where the choice of time horizon should be informed by the duration of infectiousness. Costs will also vary across settings [19], as will the functional form of the costs over time. Therefore, we should not seek a generalizable cost function or values but to develop a framework to guide that process [19].

Model predictions and effectiveness measures could be connected to program costs to evaluate the cumulative cost and health impact of resurgence or elimination under different surveillance programs. We must also consider the role that these models will have in equity of health. The optimal model for EPHP given monetary costs may suggest stopping treatment and switching to passive surveillance with a likelihood of some individuals in the population still being infected.

## CHALLENGES TO MODELING SUSTAINABLE CONTROL

Modeling sustainable control programs will entail multiple challenges. First, the mathematical models that will be used to calculate effectiveness metrics are poorly validated at low prevalence. Model validation at these low prevalence settings will need to take place, but the timescale of this validation will be at odds with the timescale for control recommendations. If a model is validated at this low prevalence, imperfect diagnostic testing may lead to difficulties in distinguishing between true elimination and false negatives. Modeling these observation processes will help accounting for uncertainty, but some outcomes, especially at low prevalence, may not be identifiable from each other.

Costs will likely be country specific, not just in terms of absolute costs but also relative to their economy. Ensuring all costs are accounted for appropriately will require collaboration with stakeholders and health economists. In addition, there may be barriers to successful surveillance or treatment that should be included in a modeling framework. For example, programs may have the drugs for treatment but do not have the resources to deliver them [19].

## Conclusions

As programs head toward the 2030 goals, transmission models will need to predict the impact of passive or reduced surveillance in low prevalence settings, quantify the effect of imperfect diagnostic testing on the dynamics of elimination, include country-specific thresholds for detection, and describe the competing costs and health impacts associated with different surveillance and responsive intervention programs.

Mathematical models and statistical methodology could have a role to play in decision making for maintaining the gains in elimination programs. Understanding the spatial heterogeneity in disease prevalence and adaptation to local circumstances will be critical for the sustainability of control measures. Newly developed statistical tools will be used quantify prevalence given limited resources and identify hotspots of transmission. In this article, we have summarized the key components and associated challenges with developing models in the next phase of NTD elimination goals (Figure 1). The optimal program will depend on the country and disease-specific goal; therefore, modelers must collaborate with the stakeholders and decision makers so that modeling can be utilized to maintain the gains.

## Notes

***Acknowledgments.*** We would like to acknowledge Diepreye Ayabina for helpful discussions.

***Supplement sponsorship.*** This article appears as part of the supplement “Sustainable control of neglected tropical diseases – surveillance and diagnostics”, sponsored by the NTD Modelling Consortium.

***Disclaimer.*** The views, opinions, assumptions, or any other information set out in this article are solely those of the authors and should not be attributed to the funders or any other person connected with the funders.

***Financial support.*** This work was supported by the NTD Modeling Consortium by the Bill and Melinda Gates Foundation (grant number OPP1184344 to A. M. and T. D. H.) and the Wellcome Trust and the Royal Society (grant number 202562/Z/16/Z to L. P.).

***Potential conflicts of interest.*** All authors: No reported conflicts of interest. All authors have submitted the ICMJE Form for Disclosure of Potential Conflicts of Interest. Conflicts that the editors consider relevant to the content of the manuscript have been disclosed.
